# A cluster randomised trial to evaluate the effectiveness of household alcohol-based hand rub for the prevention of sepsis, diarrhoea, and pneumonia in Ugandan infants (the BabyGel trial): a study protocol

**DOI:** 10.1186/s13063-023-07312-1

**Published:** 2023-04-17

**Authors:** Martin Chebet, David Mukunya, Kathy Burgoine, Melf-Jakob Kühl, Duolao Wang, Antonieta Medina-Lara, Eric Brian Faragher, Amos Odiit, Peter Olupot-Olupot, Ingunn Marie Stadskleiv Engebretsen, John Baptist Waniaye, Julius Wandabwa, Thorkild Tylleskär, Andrew Weeks

**Affiliations:** 1 Faculty of Health Sciences, Busitema University, Mbale, Uganda; 2 Centre for International Health, Department of Global Public Health and Primary Care, University of Bergen, Bergen, Norway; 3 Sanyu Africa Research Institute, Mbale, Uganda; 4 Mbale Regional Referral Hospital, Mbale, Uganda; 5 Liverpool School of Tropical Medicine, Liverpool, UK; 6 Department of Public Health and Sport Sciences, University of Exeter, Exeter, UK; 7 Ngora Freda Carr Hospital, Ngora, Uganda; 8 Mbale Clinical Research Institute, Mbale, Uganda; 9 Ministry of Health, Kampala, Uganda; 10 Sanyu Research Unit, Department of Women’s and Children’s Health, University of Liverpool, Liverpool Women’s Hospital, Crown Street, Liverpool, L8 7SS UK

**Keywords:** Sepsis, Alcohol-based hand rub, Newborn, Neonatal, Uganda, Infections

## Abstract

**Background:**

Infections are one of the leading causes of death in the neonatal period. This trial aims to evaluate if the provision of alcohol-based hand rub (ABHR) to pregnant women for postnatal household use prevents severe infections (including sepsis, diarrhoea, pneumonia, or death) among infants during the first three postnatal months.

**Methods:**

Through a cluster-randomised trial in eastern Uganda, 72 clusters are randomised in a 2-arm design with rural villages as units of randomisation. We estimate to include a total of 5932 pregnant women at 34 weeks of gestation. All women and infants in the study are receiving standard antenatal and postnatal care. Women in the intervention group additionally receive six litres of ABHR and training on its use. Research midwives conduct follow-up visits at participants’ homes on days 1, 7, 28, 42, and 90 after birth and telephone calls on days 14, 48, and 60 to assess the mother and infant for study outcomes. Primary analyses will be by intention to treat.

**Discussion:**

This study will provide evidence on the effectiveness of a locally available and low-cost intervention in preventing neonatal sepsis and early infant infections. If ABHR is found effective, it could be implemented by adding it to birthing kits.

**Trial registration:**

Pan African Clinical Trial Registry, PACTR202004705649428. Registered 1 April 2020, https://pactr.samrc.ac.za/.

**Supplementary Information:**

The online version contains supplementary material available at 10.1186/s13063-023-07312-1.

## Background

Globally, an estimated 5 million children younger than five years of age die annually [1]. The leading causes of global under-5 deaths are neonatal morbidity (37.3%), followed by lower respiratory tract infections such as pneumonia (13.3%) and diarrhoeal diseases (9.9%) [1]. While mortality after the first 28 days of life in Uganda has significantly reduced in the last two decades, reduction in neonatal mortality has been slow with an 18% reduction (from 33 to 27 deaths per 1000 live births) [2]. Infections are one of the leading causes of death in this neonatal period [3]; the common neonatal clinical conditions of sepsis, meningitis, and pneumonia account for approximately one third of all neonatal deaths in sub-Saharan Africa [4]. Reducing infant infections is therefore a prerequisite for Uganda to reduce the neonatal and under-5 mortality and therewith achieve the Sustainable Development Goal 3 (SDG target 3.2: ‘reduce neonatal mortality to less than 12 deaths per 1000 live births and under-5 mortality to at least as low as 25 per 1000 live births’) [5, 6].

Poor sanitation and hygiene amongst parents and other carers as well as a lack of proper hand-washing facilities contributes to the high burden of infections [7, 8]. In the most recent Ugandan National Household Survey, only 19% of the households used improved toilet facilities, and only 59% had a hand washing facility [7]. Furthermore, pilot work from the BabyGel scoping survey in Mbale district showed that approximately 53% of mothers do not wash their hands regularly and 47% of mothers only wash their hands when they are heavily soiled. Hand hygiene has been a high priority for the World Health Organization (WHO), emphasised in the ‘Seconds save lives: clean your hands’ global campaign since 2009 [9]. Unfortunately, in most low-income countries (LIC), compliance to hand washing even among health workers in critical medical situations is only about 10% [10]. Interventions to promote hand hygiene in LICs are needed.

The WHO campaign states that reliable uninterrupted provision of good-quality ABHR would improve hand hygiene [9]. However, there is little high quality evidence to support this advice, especially for community settings. A quasi-experimental study at Mbale Regional Referral Hospital noted that provision of ABHR in hospital wards coupled with training greatly improved hand hygiene [11]. In contrast to hospital settings, the provision of latrines, hand-washing facilities and hygiene education to communities in Zimbabwe had no effect on rates of infant infection or their gut microbiome despite the facilities being well used [12]. An intervention such as ABHR use by the caregiver that is closer to the infant may therefore be needed to break the faecal-oral transmission pathway. In this study, we aim to determine whether the community based provision of ABHR with hygiene training to pregnant women for postnatal household use is effective for the prevention of severe illness or death during the first 3 months of life.

### Study objectives

#### Primary objective

- To evaluate the effect of ABHR use by carers on severe illness or death in infants in the first 3 months of life.

#### Secondary objectives

To evaluate the effect of ABHR use by carers in infants in the first 3 months of life:

- On rates of diarrhoea
- On rates of respiratory tract infections
- On rates of omphalitis
- On rates of other infections
- On linear and ponderal growth

In addition:

- To estimate the cost of providing ABHR and assess the cost-effectiveness of providing the ABHR use versus normal practice
- To explore the effect of ABHR provision on maternal behaviour
- To evaluate the effect of ABHR use on rates of maternal sepsis

#### Exploratory objectives

- To explore the relationship between health inequalities and rates of maternal and infant morbidity
- To explore current hand hygiene practices and options of improving practice in rural Ugandan villages
- To explore the mechanisms of action (mediators and moderators) of caregiver ABHR training on prevention of severe illness or death during the first 3 months of life

## Methods

### Study design

This study is a phase 3 open-label, 2-arm, stratified cluster-randomised controlled trial. ABHR is not classified as a drug in Uganda and this is therefore not a Clinical Trial of an Investigational Medicinal Product.

### Study setting

The trial is being conducted in Mbale and Budaka Districts in Eastern Uganda, located at the foot of Mt Elgon and approximately 230 km east of the capital Kampala.

#### Hand washing practices

In Uganda, hand washing usually takes place with the midday meal when hands are washed with water before and after eating. In addition, people often wipe their hands on a cloth throughout the day. As a consequence, their hands are often free of particulate matter, allowing for alcohol-based hand rub to be effective.

#### The BabyGel pilot trial

A pilot trial was conducted on the provision of ABHR to postpartum mothers to prevent neonatal infective morbidity [13]. The average gel use over 3 months was 2.5 litres (range 1.1–4.1). There was also evidence of a behavioural effect of having the ABHR, with an increase in care-seeking behaviour in the treatment clusters [14]. Participant interviews and focus groups showed high knowledge, acceptability and compliance with the study intervention.

### Study population

Eligible participants are women who are over 34 weeks pregnant (estimated by menstrual or ultrasound scan dates or physical observation) and residing in the included 72 clusters.

#### Inclusion criteria

- Participant is willing and able to give informed consent for participation,
- Female, aged 18 years or above, or those under 18 so long as they are emancipated,
- At least 34 weeks pregnant,
- Living in one of the clusters in Mbale or Budaka Districts, defined within this study protocol, and planning to be live there for birth and the first 3 postnatal months.

#### Exclusion criteria

- Women who have previously participated in the BabyGel study and present with a further pregnancy

### Intervention

The Ugandan government encourages the public to use ABHR. ABHR has been declared a safe public health item, and the BabyGel pilot study confirmed safety, tolerability, and acceptability of the ABHR [13, 15].

#### Comparator arm

All participants receive standard antenatal care including health information regarding hand hygiene with soap and water, and a Maama birth kit (Picture 1). Following recent national recommendations, all women also receive chlorhexidine for cord care [16]. All women receive an initial visit from the research midwives at home after 34 weeks in addition to standard antenatal care at the local hospital or health centre.

#### Intervention arm

Women in the intervention arm receive all the comparator arm interventions as above. In addition, they receive a total of six litres of ABHR: a full 1-litre dispenser for use at home and an empty 60 ml-bottle for use outside of the house) and a 5-litre container for refill (Picture 2).

When the ABHR is delivered, each household obtains training on the correct use of ABHR in late pregnancy and postnatal. This is repeated at fortnightly meetings, face-to-face, or by telephone, until the participant gives birth (Table 1). During the scheduled visits, the trainer measures the amount of remaining ABHR in order to monitor usage and counsels the participant if ABHR is being underused or overused. Implementing provision of ABHR will not require alteration to usual care pathways and these will continue for both trial arms. We will discontinue the ABHR if the participant develops a reaction to the product or requests to opt out.

**Table 1:** Participant’s timeline with study procedures conducted by research midwife (RM) for recruitment and follow-up and by the ABHR trainer

	**Study period**													
	**Enrolment**	**Post-enrolment**												**Exit**
**Timepoint**	***Week 34***^a^	***Week 34***^a^	***Week 36***^a^	***Week 38***^a,b^	***Week 40***^a,b^	***48 hours***	***Week 1***	***Week 2***^b^	***Week 4***	***Week 6***	***Week 8***^b^	***Week 10***^b^	***Week 12***	***Week 12***
**Enrolment:**														
**Eligibility screen**	X													
**Informed consent**	X													
**Interventions:**														
***Training on ABHR use***		X	X	X	X	X								
***Assessment of ABHR use***			X	X	X	X								X
**Assessments:**														
***Baseline data collection***	X													
***Clinical, AE and SAE assessment, anthropometry***		X	X	X	X	X	X	X	X	X	X	X	X	
***Outcome assessment***						X	X	X	X	X	X	X	X	

^a^If still pregnant

^b^Telephone visit

A double-sided poster (Appendices 1 and 2) supplements the training, summarising the instructions in both a written and visual format, with one side specifying the correct antenatal and one the correct postnatal use of ABHR. The key message is that everyone should use ABHR “before touching the baby” (Appendix 3).

#### Trial organisation

For ease of study implementation, the study area is divided into three geographical areas each consisting of a minimum of 24 clusters, each with a study management site (‘hub’) hosted at the largest government run health facility in the area, in Busiu Health Centre (HC) IV, Budaka HC IV, and Kolonyi HC III, respectively. Each hub has a study doctor, village health team members and supervisors, a data manager, at least 7 research midwives to collect data, and 3 staff to train mothers in ABHR use. A cluster is eligible for inclusion in the study if the 1–4 village(s) constituting it have more than 600 inhabitants collectively and if it is not directly neighbouring another cluster (to prevent communication and intervention contamination). The aim is an average cluster size of 850 inhabitants. We surveyed each participating cluster prior to the initiation of the study to determine the population of children under the age of 2 years as a proxy for that cluster’s birth rate. In total, we expect to recruit 5932 pregnant women. Consent was obtained from the leaders of all included villages to approach and recruit mothers potentially eligible for inclusion in the study.

#### Randomisation

The randomisation process was conducted in each of the three hubs separately and in the presence of representatives from each village within that geographical area. The clusters in each area were listed in order of their population size. The four clusters with the largest populations were labelled “stratum 1”, the next four largest as “stratum 2”, and so on down to the four smallest size clusters (stratum 6). Two red and two yellow balls were put in an opaque cotton bag. The representatives from stratum 1 were called forward and each in turn blindly withdrew one ball without replacement. This process was repeated for the remaining five strata in turn to randomly designate 12 clusters as “red” and 12 as “yellow”. Finally, just one red and one yellow ball were placed in the bag and the eldest of the representatives present asked to blindly withdraw one ball. The clusters designated to the colour of the chosen ball were allocated to the intervention arm of the study and the clusters designated to the colour undrawn were allocated to the comparator arm of the study. The randomisation was thus stratified by geographical area and by population size. EBF, a researcher not involved in the day-to-day running of the trial has, kept the final randomisation sequence and will only avail this to the other scientists at the end of trial.

### Identification, recruitment, and study procedures

The study processes were initially piloted in 10 villages [13] and refined, resulting in this final procedure. The village health team (VHT) member identifies potential participants and inquires whether they are interested in being enrolled in the study. Women may also be identified when presenting at antenatal clinics in hub facilities. The VHT notifies the research midwife about the potential participant. The research midwife visits the home and spends time with the woman explaining the study, what is involved, how long the woman and her baby are involved in the study and whether she is willing to give informed consent to participate. Each woman is told that once she has given her consent, she is free to withdraw from the study at any point and that the care and treatment of herself and her baby will not be affected. Should a woman withdraw from the study, the research team gently question her to attempt to ascertain the reason for her withdrawal from the study. The same is done for women who decline to enrol. No pressure is exerted should the woman be resistant to giving a reason, but feedback obtained from this interaction provides valuable feedback to the research team for approaching other women in the study.

Those in the intervention arm are given their supply of ABHR and trained fortnightly in its use by the ABHR trainer in a series of antenatal visits and reminder calls. Those in the control arm receive only a single recruitment visit antenatally in addition to standard antenatal care.

A VHT member (or the woman herself) is encouraged to inform the BabyGel midwives or hotline (calls are free of charge) when she has given birth and a research midwife visits within 48 h either at the participant’s home or at a health facility if the participant is still admitted. The same research midwife performs regular telephone calls and personal follow-up visits up to 12 weeks (trial completion).

The women are screened and recruited into the study at 34 weeks’ gestation. Gestational age is measured using an application on the midwives’ tablet or the obstetric ultrasound report if available or physical inspection. The women and their babies are followed for 3 months post-delivery. Therefore, participating women completing the follow-up period will be active in the study for approximately 20 weeks (± 2 weeks to account for inaccurate gestational age estimations).

Mothers are informed that, should their baby become sick for any reason, they can notify the study hotline, or beep/flash the BabyGel team or VHT, or come straight to the health facility. She and/or her baby are given free treatment and reimbursed travel expenses, a small daily subsistence payment is paid for any night that the mother and/or baby is admitted. Research midwives or VHTs refer babies with signs or symptoms of sickness to the nearby health facility or the Mbale Regional Referral Hospital Neonatal Unit (MRRH NNU) for specialised care. Study doctors are trained in the study protocol and to collect standardised data. They review the sick babies and either treat or transfer to MRRH NNU according to local guidelines and standard operating procedures.

As in the pilot study, all data are collected on REDCap software (Vanderbilt University, USA) installed on tablet computers and transferred to the Clinical Trial Unit (CTU) at the Liverpool School of Tropical Medicine through an encrypted transfer.

### Masking and code breaking

Although this is an open-label study, we attempt to mask the research midwives during the follow-up period (the trainers cannot be blinded as they deliver the ABHR to the participants). This is achieved by separating the research midwives and the trainers in two separate teams in different locations. Furthermore, we ask the study participants not to discuss their hand hygiene methods with the research midwives, and the mother is asked to keep the ABHR bottles out of sight of the research midwives on the day of the face-to-face visit. A research midwife informs the mother of the physical visit in advance to enable her to hide the ABHR if in an intervention cluster. Research midwives are encouraged to hand wash with soap and water in all clusters to minimise contamination. At the end of the 3-month follow-up, the closeout interview (which includes questions on the ABHR use and acceptability) is conducted by the trainer to prevent un-blinding of the research midwife to the village allocation.

### Study assessment visits

Table 1 shows the time schedule for study procedures conducted by research midwives and ABHR trainers.

### Safety reporting

Safety of the women and babies participating in this study is paramount. Although this is not a clinical trial of an investigational medicinal product, safety data on both the women and the babies (including adverse events (AEs) and serious adverse events (SAEs) are collected carefully and reported to the sponsor, local IRB, and the Independent Safety and Data Monitoring Committee (ISDMC). The key participants in this study are the neonates, and adverse events that could be influenced by the trial interventions are outcomes for the study. Data on these events are recorded on the electronic Case Report Forms (CRFs).

### Data management

Data capture is primarily on electronic case report forms that were created using the REDCap software package (Vanderbilt University, USA) on dedicated study tablets. All records are stored and backed-up on a continuous basis on a secure off-site server at LSTM on an encrypted standalone hard drive. The data manager and information systems developer are based in the GHTU and are responsible for management of these data after they are transferred to Liverpool. Three hub data supervisors oversee each of the three study centres (hubs) in Mbale and Budaka, each of whom are responsible for curating data collected at their centre; the team work closely with the GHTU and meet weekly to reconcile any anomalies. The research data will be stored long-term in its original electronic format, both in a unified large database and in a public database containing all data collected during the study except for any information that could identify individual mothers or their babies. The public database will be updated as necessary to achieve long-term preservation (e.g. should software becomes obsolescent). These data will be preserved for 15 years. On the consent form, participants are asked if they agree to the use of their data for future research or studies without notifying them. Participants are also asked for permission for the research team to share relevant data with people from the Universities taking part in the research or from regulatory authorities, where relevant. This trial does not involve collecting biological specimens for storage.

### Statistical methods

#### Size determination

The total sample size for this study is 5932 mothers and their babies (multiple births were considered to be a single birth for this calculation). The sample will be obtained from 72 clusters arranged in 3 geographical areas of 24 clusters. The intra-cluster coefficient (ICC) estimate from the pilot study was 0.17 (95% CI 0 − 0.65); however, a study by Pagel [17] reported ICC estimates from five similar context cluster-randomised trials predominantly in the range 0.01 to 0.10, which is considered more realistic for the proposed study. The sample size calculations are based on a primary endpoint of severe infection, with estimated rates of 5–30%.

The anticipated sample size will have:

- 90% power to detect a reduction in infection rate of ≥ 25% for comparator arm rates down to 15% if ICC ≤ 0.01, or to detect a reduction in infection rate of ≥ 33% for comparator arm rates down to 5% if ICC ≤ 0.001.
- 80% power to detect a reduction in infection rate of ≥ 25% for comparator arm rates down to 10% if ICC ≤ 0.01, or to detect a reduction in infection rate of ≥ 33% for comparator arm rates down to 5% if ICC ≤ 0.005.

In the pilot study, we had a small (2%) loss to follow-up rate. Therefore, only complete cases will be used with no imputation of missing outcomes. No adjustment of sample size is planned for such a small rate of loss to follow-up, but the rate is being monitored by the ISDMC so that the final recruitment time can be extended if needed.

Clusters that reach their planned sample size early will continue to recruit until the study recruitment period is completed. If the planned recruitment size is not achieved by the end of the planned time period, the study may be underpowered, in which case permission will be sought from the management committees and funder to continue recruiting for a longer period to reach the required sample size.

Should it prove impossible to select 72 village clusters that meet the above selection criteria and have a combined estimate of 5932 births over the study period, additional clusters will be recruited into the study as feasible, to achieve the required sample size. If, however, the projected birth rate across the area covered by the three study areas falls below the required level and additional clusters are needed during the study period, the selection of additional clusters will be carried out in conjunction with the ISDMC. To preserve the balance in the two study arms, clusters will be recruited into a study area in pairs; one cluster in each pair will be allocated to the intervention arm and the other in the pair to the control arm.

#### Statistical analysis plan

As some mothers in clusters randomised to the intervention arm may not use the ABHR provided while others in clusters randomised to the comparator arm may acquire an ABHR for themselves, the primary statistical analysis will be by the principle of “intention-to-treat (ITT)”. That is, all mothers and babies will be analysed in the study group to which their village of residence was randomised; this provide appropriate allowance for non-compliance in both groups and produce estimates of intervention effect that are more reflective of what might occur should the provision of an AHBR to new mothers be adopted as a health policy.

Initially, incidence rates of the primary outcome will be compared across the two study arms using generalised estimating equations (GEEs) to allow for clustering effects. A negative binomial distribution and log link function will be used with outcomes in the same cluster assumed to be equally correlated (exchangeable). Effects sizes will be reported as incidence rate ratios (IRRs) with their 95% confidence intervals calculated using robust Huber-White “sandwich” standard errors to correct for any slight misspecification of the correlation structure. Time to the first occurrence of the primary outcome then be analysed in the same way but using Cox proportional hazards regression model with frailty at village level with mothers/babies lost to follow-up included as censored observations. Intervention effects will be reported as hazard ratios with their (robust) 95% confidence intervals. IRR and hazard ratio estimates will be presented both unadjusted and adjusted for important covariates (including age of mother, sex of infant, study hub/geographical area, WASH status (availability of tapped water and type of latrine in dwelling), rural and peri-urban status, highest education level achieved by mother).

All secondary outcome measures will be assessed also using GEE techniques as for the primary outcome, with appropriate distributional assumptions depending on the statistical nature of each measure. Effect sizes will be presented with their (robust) 95% confidence intervals, both unadjusted and adjusted for important covariates. However, to gain some insight into the possible effect of ABHR if adopted fully, a secondary analysis will be performed on a per protocol principle.

Missing baseline covariates will be imputed using simple imputation methods in the covariate-adjusted analysis based on the covariate distributions. For a continuous variable, missing values will be imputed from random values from a normal distribution with mean and SD calculated from the available sample. For a categorical variable, missing values will be imputed from random values from a uniform distribution with probabilities P1, P2, …, and Pk from the sample. For a count data, missing values will be imputed from random values from a Poisson distribution with λ from the sample. Seed for the imputation is set as 128.

Due to the nature of the study, no stopping rules have been set. Any decision to stop the study will be based on a combination of rates of adverse events, recruitment rate, interim data analysis, and the results of other recently published studies. The final decision to stop the study would be made by the trial steering committee.

### Economic evaluation

An economic evaluation will be conducted alongside the RCT to estimate the cost and cost-effectiveness of the intervention versus the control; analyses will be based on intention-to-treat (ITT) in line with the statistical analysis. We will estimate the cost-effectiveness of the intervention based upon the primary effectiveness outcome (severe illness or death in infants in the first 3 months of life). We will use a societal perspective and include a broad range of costs including (but not only) out-of-pocket costs on antibiotics by the household. The resources used to deliver the intervention are collected from the study sites using a micro-costing approach and the Cost of Integrated Newborn Care (COIN) framework and include set up and training costs so that policy-makers are aware of the resource implications of adopting this intervention. Face-to-face standardised questionnaires are used to collect information on socio-economic status of households and health-seeking behaviour and health resource utilisation at baseline and 3 months. A regression model will be used to adjust for systematic differences between intervention and placebo arms at baseline and will be used to calculate the incremental cost-effectiveness ratio (ICER) for the primary outcome measure. A bootstrap procedure to estimate the confidence intervals of the ICER estimates and cost-effectiveness acceptability curves will be used to describe the likelihood of cost-effectiveness at different cost-effectiveness thresholds from WHO [18, 19]. The robustness of the results will be assessed through one-way sensitivity analyses and by using a Tornado diagram to show the greatest sources of uncertainty. All prices in Ugandan shillings will be exchanged to Purchasing Power Parity US dollars in order to allow international comparability.

### Quality assurance procedures

The study is monitored (by the Sponsor) in accordance with the current approved protocol, GCP, relevant regulations, and standard operating procedures. Monitoring occurs once a year. Concise SOPs for each of the study procedures are present. The REDCap data collection system includes a facility to prevent the input of incorrect data and to check unlikely data values.

### Participant and public involvement

We involved the women, families, and the public from the participating sites in the study design and continue to engage them in the conduct of the BabyGel trial. A participant and public involvement (PPI) steering team oversees all aspects of PPI in the project, including study design, trial information, protocol, and data collection tools or any other study specific documents, analysis, reporting, and dissemination. They also provide oversight for the conduct of PPI design workshops or group discussions with participants, mothers, caregivers, husbands, and clinicians.

We set up a community advisory board of 10 members at each hub. These comprise a pastor, imam, local council representative, facility in charge, a facility midwife, a private clinic representative, a mothers’ representative, father representative, a school teacher, mother-in-law, and elderly woman. These meet quarterly and discuss the project, review study protocol, documents, plans, questionnaire, progress, and interpret study outcomes.

The PPI steering team has established a specific local PPI groups at each hub, namely;

1. Budaka hub: the sick mother/ baby group in the intervention arm,
2. Busiu hub: the sick mother/baby group in the comparator arm
3. Kolonyi hub: Non-study participants with sick-babies or sick mothers themselves

These serve as local maternal and infant infection support groups in the area. At each time, the composition is about 10 members. These groups provide a forum through which the trial team can engage with users and ensure responsive feedback. The feedback from these groups is communicated to the site trial management team (site TMT), the international trial management group (TMG), and trial steering committee (TSC).

### Trial management

The study has four oversight committees:

- Trial management group (TMG)

This group consists of the CI (as the chair), study statistician, programme manager, GHTU manager, and work package leads, together with representatives from the partner organisations. This group takes responsibility for the day-to-day operational issues in delivering this study to time and target. The group meets at least once monthly in accordance with the TMG charter at which hubs will summarise progress and challenges and bring up for discussion any difficulties, as well as discuss and decide matters of general importance for the trial. Due to geographic distances between the partner organisation meet via zoom but the group tries to meet face-to-face at least once per year around the time of a national or an international conference or other meeting. All decisions regarding the overall running of the trial are made in this forum with the exception of matters of fundamental importance to the viability of the trial or that require major changes to the protocol. These will be referred to the trial steering committee (TSC).

b) Trial steering committee (TSC)

This group has overall executive decision-making powers in relation to running of the study and has strategic responsibility for the study in line with its charter. The group consists of three members completely independent to the study together with two members, the CI and the study statistician, together with the sponsor’s representative. Due to geographic distances between the partner organisations, this meeting will be via teleconference/SKYPE, but the group will try to meet face-to-face at least once during the study. A charter will be developed to describe the functioning of the TSC.

iii) Independent Safety and Data Monitoring Committee (IDMC)

The committee (ISDMC) comprises one neonatal clinical trials-experienced clinician, one hand hygiene research experienced public health expert, one health economist, and a statistician, none of whom have direct involvement with the study. The IDMC reports to the TSC. The main responsibilities of this committee is to safeguard the interests of trial participants, potential participants, investigators, and sponsor, to assess the safety and efficacy of the trial’s intervention, to monitor the trial’s overall conduct, and to protect its validity and credibility.

iv) Trial management team (TMT)

This group meets monthly and is chaired by the principal investigator or co-principal investigator at the site. The group discusses issues related to the progress of the trial at the site, and to ensure that the trial is running well.

### Dissemination

The results from the various work packages will be published as soon as possible. All papers will be submitted to high-level open access health journals. The Uniform Requirements for Manuscripts Submitted to Biomedical Journals (http://www.icmje.org/) and the CONSORT checklist for cluster-randomised trials will be used to assist with clear reporting. The trial management group forms the basis of the writing committee for the main study and gives advice on the publication and authorship of all other publications. Local dissemination meetings will be held in Kampala, Mbale, and Budaka for policy-makers and staff and in Mbale and Budaka districts for the public including local participants in the study.

## Discussion

We are evaluating whether the provision of alcohol-based hand rub (ABHR) to pregnant women for postnatal household use is effective for the prevention of severe illness or death during the first 3 months after birth. To the best of our knowledge, this is the first study that determines the effectiveness of ABHR given to mothers in preventing infant infections. Our intervention has high potential for scale-up as the ABHR can be added to birth kits that are routinely given to pregnant women. Furthermore, ABHR used in this trial was locally manufactured from a sugar processing plant and is therefore financially sustainable. The COVID-19 pandemic normalised the use of ABHR also among persons who previously showed scepticism given the alcoholic nature of the product.

Unlike previous trials in LICs that assessed whether provision of pit-latrines and hand washing facilities reduces infections, our study assessed a more proximal intervention; as the use of ABHR before touching the infant could prevent transfer of infections to the infant whether or not the family has a pit latrine. ABHR potentially reduces the inconveniences encountered in implementing hand hygiene in complex household situations where water is often scarce. Also, in places where hand washing is not feasible, practical or convenient such as funerals, parties, and while travelling, ABHR is preferable.

The large size of the trial reduces the likelihood of a type II error. The main study outcome is validated by a group of three medical doctors (a neonatologist, a paediatrician and a general doctor) and as such the likelihood of measurement bias is reduced. Also, since our definition of sepsis involves hospitalisation or death, which are hard outcomes, we reduce the potential of measurement bias since some of the signs and symptoms of child infections are non-specific.

Regular visits by trained midwives will most likely reduce the incidence of infection and death, due to early detection of poor practices or signs of infection and initiating the necessary referral. Also, the availability of study doctors and medication ensures that participants in the trial get superior care to the rest of the infants. Finally, COVID-19 normalised ABHR use; as such, participants in the control villages could use it as measure against COVID-19. We collect data on its use in both arms and will do a per-protocol analysis to assess this effect.

## Trial status

Recruitment started on 11 January 2021 and is on-going. Anticipated completion date is April 2024.

## Supplementary Information

**Additional file 1: Appendix 1.** Poster shown and given in the antenatal period to supplement alcohol based hand rub training in the BabyGel trial.

**Additional file 2: Appendix 2.** Poster shown and given in the postnatal period to supplement alcohol based hand rub training in the BabyGel trial.

**Additional file 3: Appendix 3.** Alcohol based hand rub training guide used in the BabyGel trial.

**Additional file 4.**

**Additional file 5.**

**Additional file 6: Picture 1.** Components of a Maama birth kit given to pregnant women in the BabyGel Trial. **Picture 2.** Alcohol Based Handrub Package given to pregnant women in the intervention arm of the BabyGel Trial.

## Abbreviations

CI: Confidence interval
ABHR: Alcohol-based hand rub
AEs: Adverse events
COIN: Cost of Integrated Newborn Care
COVID-19: Coronavirus disease 2019
CRF: Case report form
GEE: Generalised estimation equations
HC: Health centre
ICC: Intra-cluster coefficient
ICER: Incremental cost-effectiveness ratio
IRB: Institutional Review Board
IRR: Incidence rate ratios
ISDMC: Independent Safety and Data Monitoring Committee
ITT: Intention-to-treat
LIC: Low-income countries
PPI: Participant and public involvement
RCT: Randomised controlled study
SAE: Serious adverse event
SD: Standard deviation
Site TMT: Site trial management team
TMG: The international trial management group
TSC: Trial steering committee
VHT: Village health team
WHO: World Health Organization
