# Supplementary material for: A cluster randomised trial to evaluate the effectiveness of household alcohol-based hand rub for the prevention of sepsis, diarrhoea, and pneumonia in Ugandan infants (the BabyGel trial): a study protocol
Source: Trials. 2023 Apr 17;24:279. doi: 10.1186/s13063-023-07312-1 (PMC10106319; doi:10.1186/s13063-023-07312-1)
Supplement: Supplementary file 1 — Additional file 1: Appendix 1. Poster shown and given in the antenatal period to supplement alcohol based hand rub training in the BabyGel trial. [file 13063_2023_7312_MOESM1_ESM.pdf]

BabyGel

BEFORE birth

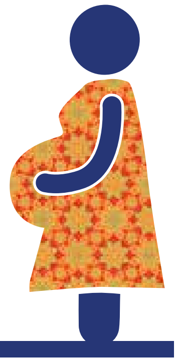

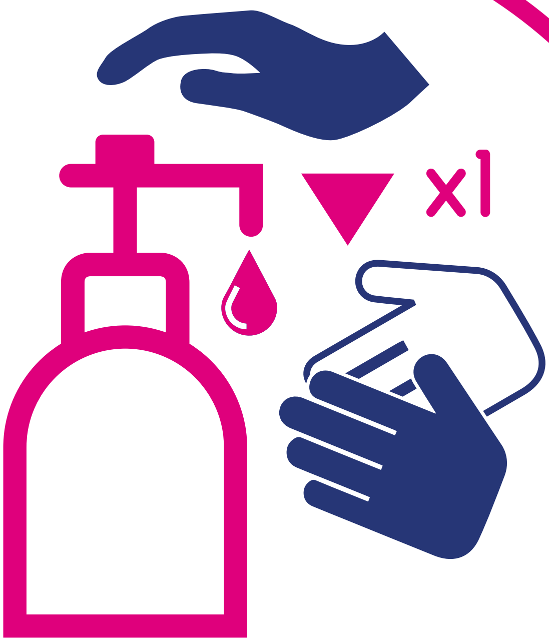

Push down once
Rub hands until dry

MOTHER ONLY

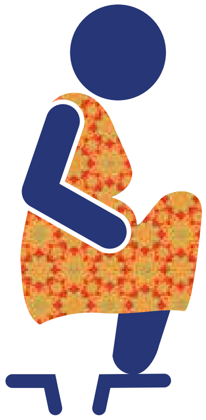

Clean hands
with BabyGel
after toilet use

EVERYONE AT TIME OF BIRTH

Clean hands
with BabyGel
before touching
mother or baby

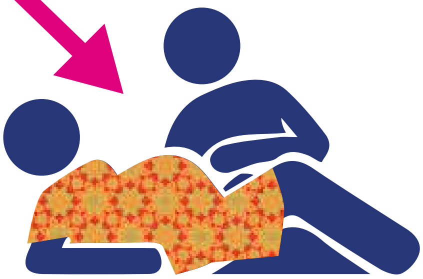

This project is part of the EDCTP2 programme
supported by the European Union
