# Supplementary material for: A cluster randomised trial to evaluate the effectiveness of household alcohol-based hand rub for the prevention of sepsis, diarrhoea, and pneumonia in Ugandan infants (the BabyGel trial): a study protocol
Source: Trials. 2023 Apr 17;24:279. doi: 10.1186/s13063-023-07312-1 (PMC10106319; doi:10.1186/s13063-023-07312-1)
Supplement: Supplementary file 2 — Additional file 2: Appendix 2. Poster shown and given in the postnatal period to supplement alcohol based hand rub training in the BabyGel trial. [file 13063_2023_7312_MOESM2_ESM.pdf]

BabyGel

AFTER baby is born

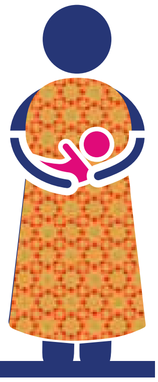

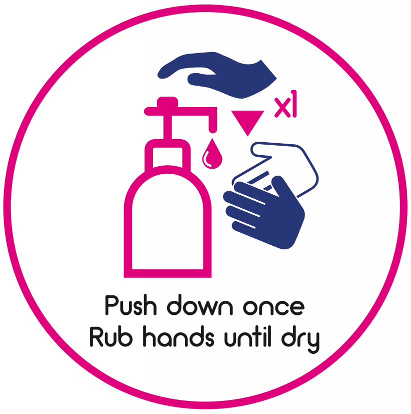

Clean hands with BabyGel after changing

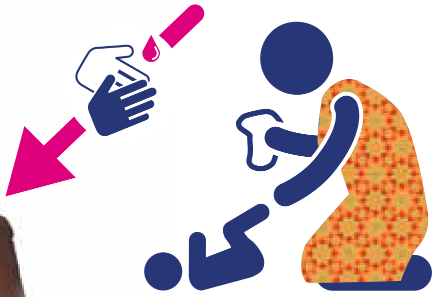

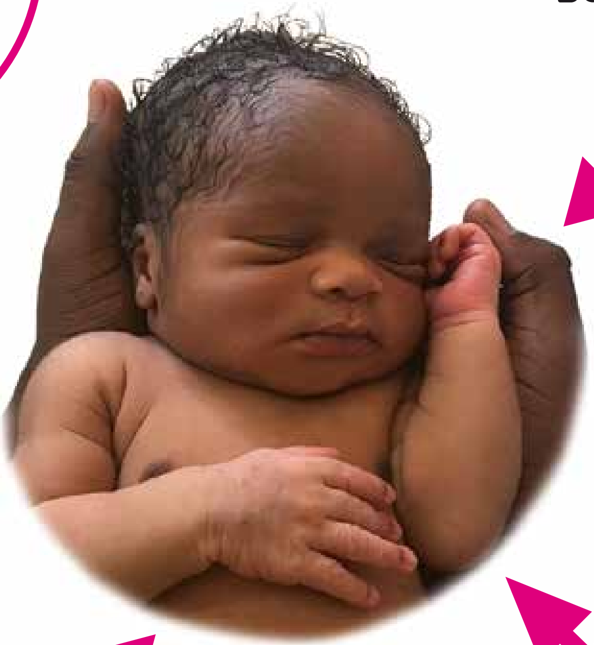

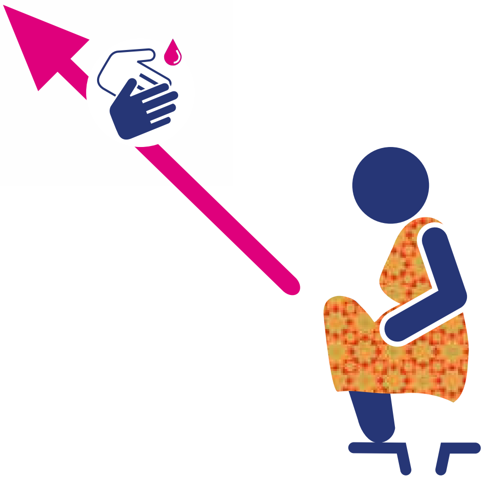

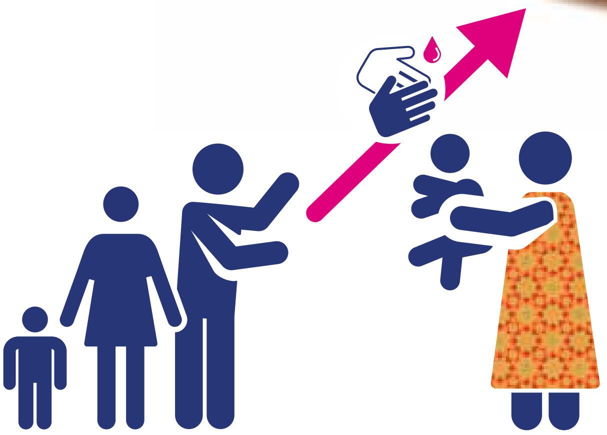

Everyone always clean hands before touching the baby

Clean hands with BabyGel after toilet use

This project is part of the EDCTP2 programme
supported by the European Union

EDCTP

SAfRI
Sanyu Africa Research Institute
