# Supplementary material for: A cluster randomised trial to evaluate the effectiveness of household alcohol-based hand rub for the prevention of sepsis, diarrhoea, and pneumonia in Ugandan infants (the BabyGel trial): a study protocol
Source: Trials. 2023 Apr 17;24:279. doi: 10.1186/s13063-023-07312-1 (PMC10106319; doi:10.1186/s13063-023-07312-1)
Supplement: Supplementary file 3 — Additional file 3: Appendix 3. Alcohol based hand rub training guide used in the BabyGel trial. [file 13063_2023_7312_MOESM3_ESM.docx]

**ABHR Trainer Guide**

**Trainer: ____________________**

**Phone:**

IMPORTANT PHONE NUMBERS:

-

-

-

-

-

**Before you leave the office to all visits …**

**… Remember to always take with you these information:**

- Contact Details of Mothers to be visited:
  - Name____________________; Phone____________________
  - Name____________________; Phone____________________
  - Name____________________; Phone____________________
  - Name____________________; Phone____________________
  - Name____________________; Phone____________________
  - Name____________________; Phone____________________
- Contact Details of Village Health Teams, LSC1 Chairman, mother
  - Name____________________; Phone____________________
  - Name____________________; Phone____________________
  - Name____________________; Phone____________________
  - Name____________________; Phone____________________
- GPS coordinates for all planned stops:

**… Remember to take these documents:**

- This Training Guide including your personal notepad
- Project ID
- Letters of permission from the district officials (RDC and DHO) and from the Uganda National Council of Science and Technology
- Identification badge
- Map of the area

**… Remember to take this equipment:**

- Charged and credit-loaded telephone, charger
- Charged tablet, charger
- Charged external battery
- Enough nails, hammer
- Food and water; or food allowance for the trip
- ABHR-kit (6l plus travel bottle) for each planned visit
- Your Training Chart
- An eraseable pen for your checklists
- 3 Pens
- Helmet if motorcycle travel is planned
- Emergency contacts of Quality Assurance Officer (QAO), Medical Officer (MO), Trial Manager (TM) and Principal investigator (PI)

**… Remember to Confirm:**

- The specific checklist for each specific visit you plan today

**VISIT 1**


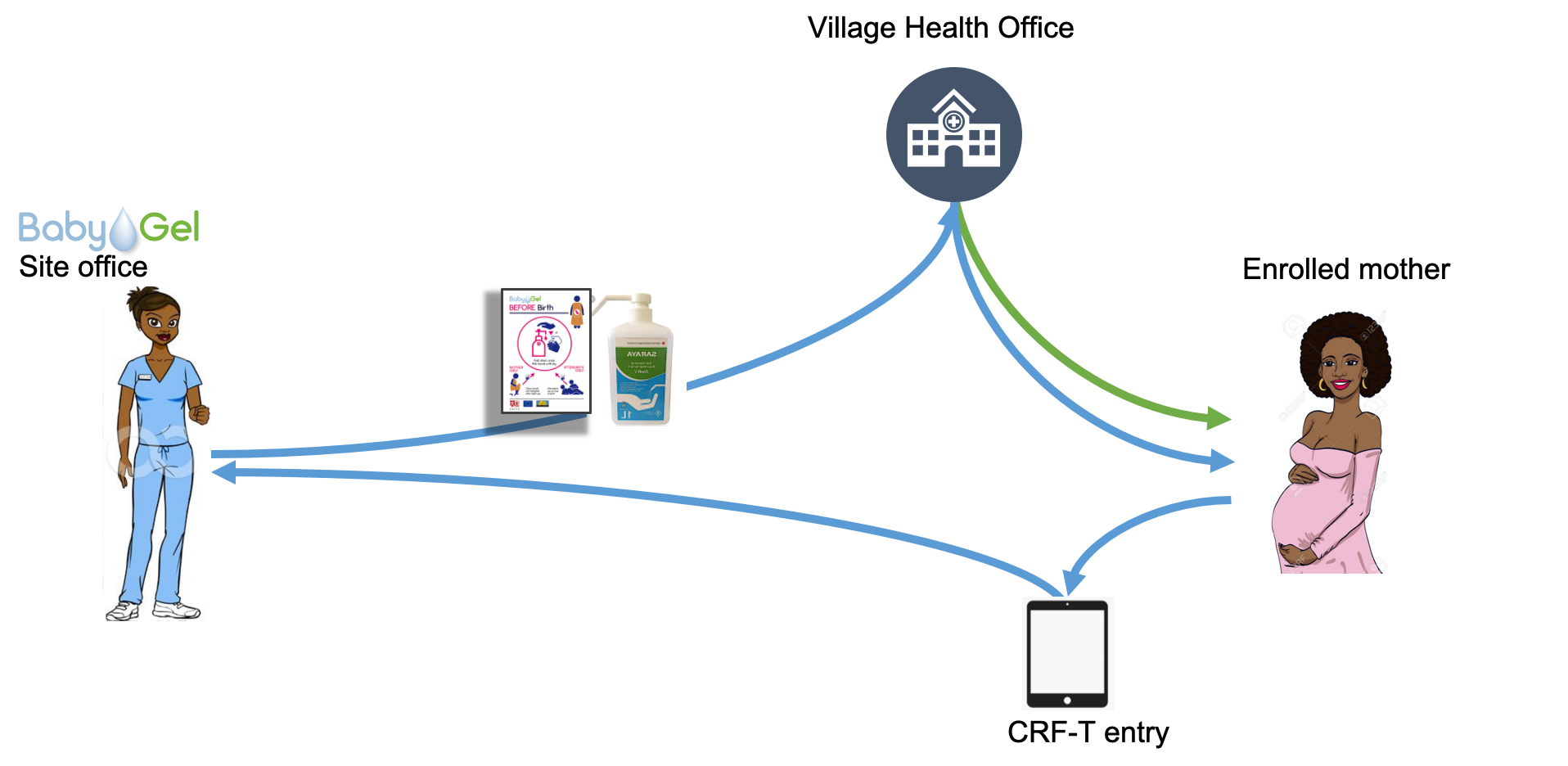


**Before you leave the office to VISIT 1:**

**Remember to take with you:**

- one laminated BabyGel Poster per pregnant woman with string attached
- one small (empty), medium, and large (full) BabyGel Container per mother in a concealed backpack
- the required accountability log for the mother

**Remember to Confirm:**

- the village is meant to receive ABHR
- Enrollment-visit with VHT and Data Collector was logged as completed
- Maama Kit was handed over to woman
- 5l container has liter-marks

**Before Training**

- **Meet Village Health Worker (or LSC1 Chairman)**
  1. Confirm name and location of pregnant woman
  2. Confirm that he/she will leave after the introduction
- **Let VHW (LSC1 Chairman) guide you to the household and introduce you**
- **Introduce yourself to woman, explaining in detail your job as trainer, and who to contact with any questions regarding the study.**

**Training**

- Invite Woman (and husband) to select training **location**
- Introduce the **content** of today, and give overview of training schedule
  - 1. Today (30-60 min.)
    2. Before and after pregnancy (2X2 visits plus phone calls)
- State **importance of hand hygiene** with water and soap, or with ABHR
- First **demonstration** of ABHR
  - 1. Presentation of 3 containers: fill small bottle with ABHR, using the 1L-dispenser
    2. rubbing demonstration, let participants try, repeat.
- Presentation of **Poster**, Explaining every detail (10 min.)
  - 1. *Before Birth*, ***mother*** can protect the baby:
       - Hands become especially dangerous after toilet use, baby changing, handling animals. Cleaninig hands is crucial for child health
       - Mother must clean hands with ABHR after toilet use
    2. *After Birth*, ***everyone*** can protect the baby:
       - The new-born baby is especially at risk of infection
       - Mother: Keep cleaning your hands after toilet use and changing the baby
       - Mother: Always use ABHR before touching the baby
       - Everyone: always uses ABHR before touching the baby
- **Practice** for each side of the poster, make scenarios, explain, test understanding
- **Repeat** until participant(s) are clear on *how* and *when* to use ABHR
- Explain refill (fill) and **safe storage** instructions
- **Confirm safety** is understood; together identify locations dispenser and container, and fix poster on the wall
- Discussion, **Q&A**, with participant(s)
- **Repetition**: Mother explains and demonstrates ABHR use to a relative (or you)
- **Correction if Needed, Final Q&A**
- *Sign Accountability Log, Reminder of next visit schedule*
- **Farewell**

**After Training, near the house**

- **Fill CRF T,**
- **Note household specific thoughts for follow-up in your pad**

**VISIT 2**

2^nd^ visit before birth

**
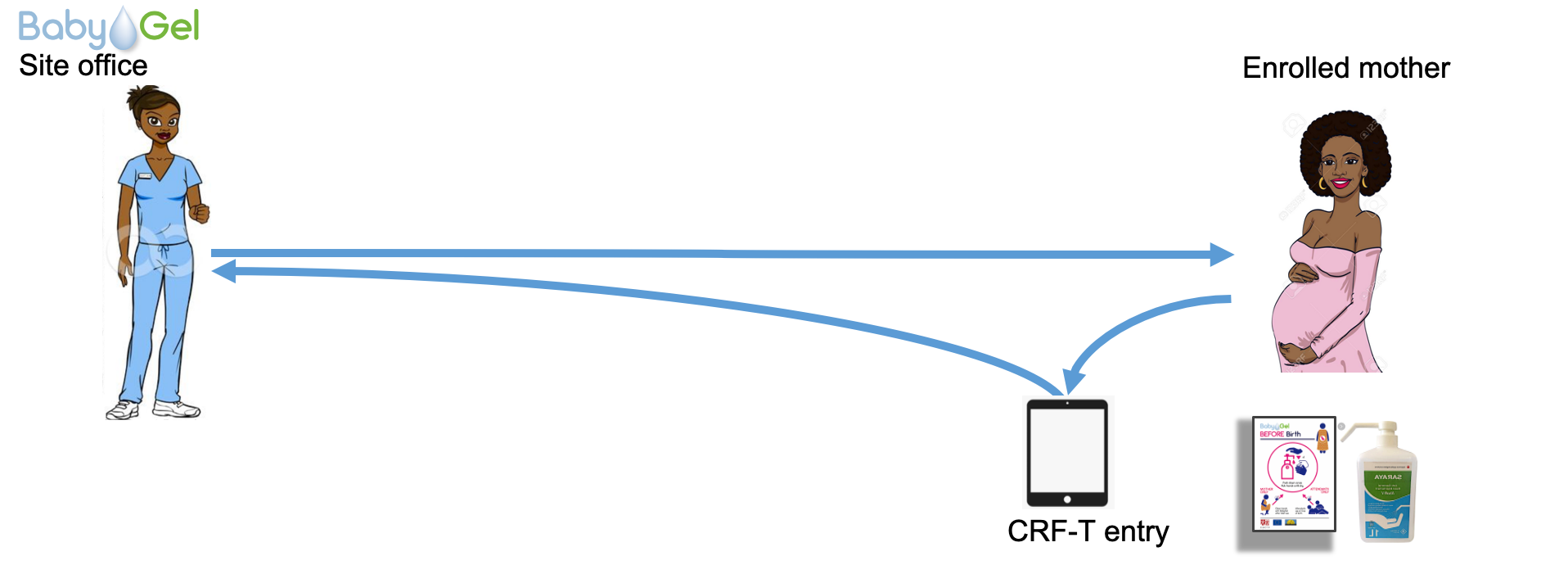
**

**Before you leave the office to VISIT 2:**

**Remember to take with you:**

- One spare poster fitted with a string
- One spare 1-L dispenser of ABHR for the day

**Remember to Review:**

- Your notes from the last visit and prepare for your action points

**Before Training**

- **Greeting** participant(s)

**Training Introduction**

- Invite participant with baby (and husband) to select training **location**
- Introduce the **content** of today, and give overview of remaining training schedule

**Training Repetition (Mother as Instructor)**

- Ask mother to fetch the dispenser and poster
- Ask mother why **hand hygiene** with water and soap, or with ABHR, is important. Correct if needed.
- **Ask mother to demonstrate** how to use ABHR
- Ask mother to explain **poster**, step by step, side by side (10 min.), correct if needed.
- **Practice** for each side of the poster, make scenarios, explain, test understanding
- **Repeat** until participant(s) are clear on *how* and *when* to use ABHR
- Ask mother to explain refill (fill) and **safe storage** instructions
- **Confirm safety** is understood; together identify locations dispenser and container, and fix poster on the wall
- Discussion, **Q&A**, with participant(s)
- **Repetition**: Mother explains and demonstrates ABHR rubbing to a relative (or you), they repeat rubbing. Mother corrects until correct.
- *Reminder of next visit schedule*
- **Farewell**

**After Training, near the house**

- **Fill CRF T,**
- **Note household specific thoughts for follow-up in your pad**

**VISIT 3**

1^st^ visit after birth

**
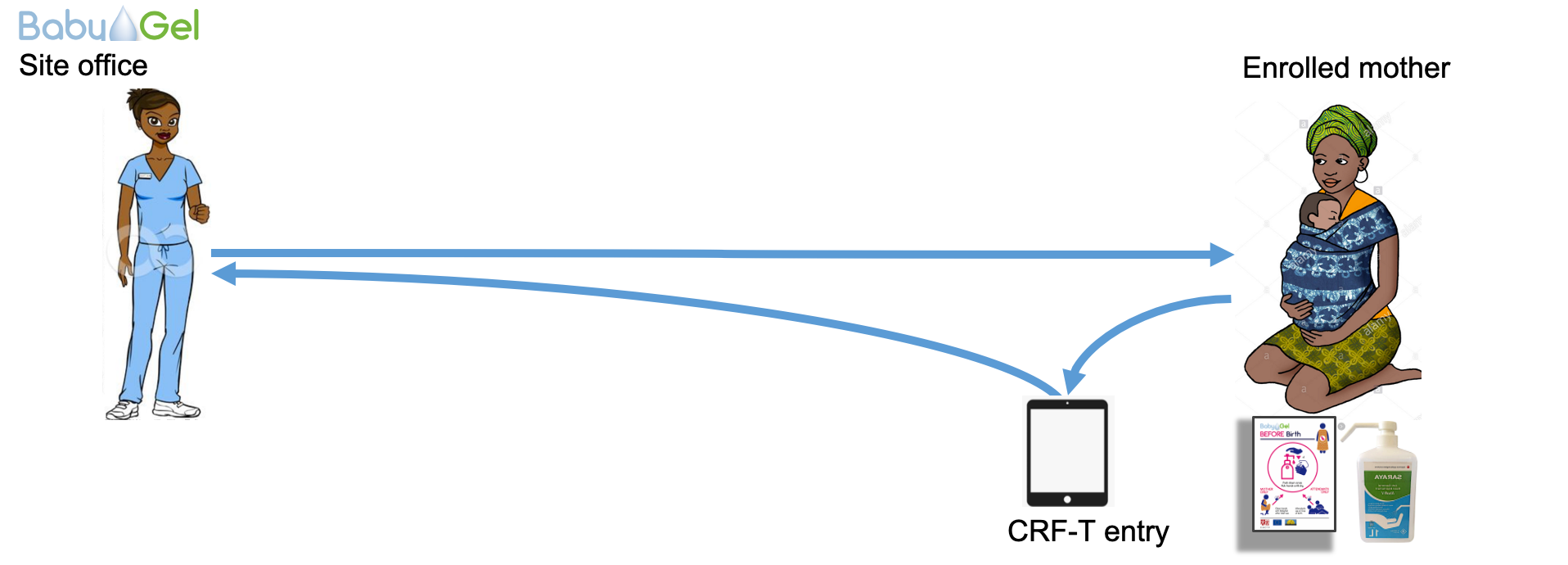
**

**Before you leave the office to VISIT 3:**

**Remember to take with you:**

- One spare poster fitted with a string
- One spare 1-L dispenser of ABHR for the day

**Remember to Review:**

- Your notes from the last visit and prepare for your action points

**Before Training**

- **Greeting** participant(s)

**Training Introduction**

- Invite mother (and husband) to select training **location,** making sure the mother is comfortable and safe.
- Introduce the **content** of today, and give overview of training schedule

**Training Repetition (Mother as Instructor)**

- Ask mother or someone to fetch the dispenser and poster
- Ask mother why **hand hygiene** with water and soap, or with ABHR, is important. Correct if needed.
- **Ask mother to demonstrate** how to use ABHR
- Ask mother to explain **poster**, post natal side (10 min.), correct if needed.
- **Practice** rubbing, make scenarios, explain, test understanding
- **Repeat** until participant(s) are clear on *how* and *when* to use ABHR
- Ask mother to explain refill (fill) and **safe storage** instructions
- **Confirm safety** is understood; together identify locations dispenser and container, and fix poster on the wall
- Discussion, **Q&A**, with participant(s)
- **Repetition**: Mother explains and demonstrates ABHR rubbing to a relative (or you), they repeat rubbing. Mother corrects until correct.
- *Reminder of next visit schedule*
- **Farewell**

**After Training, near the house**

- **Fill CRF T,**
- **Note household specific thoughts for follow-up in your pad**

**VISIT 4**

Last visit

**
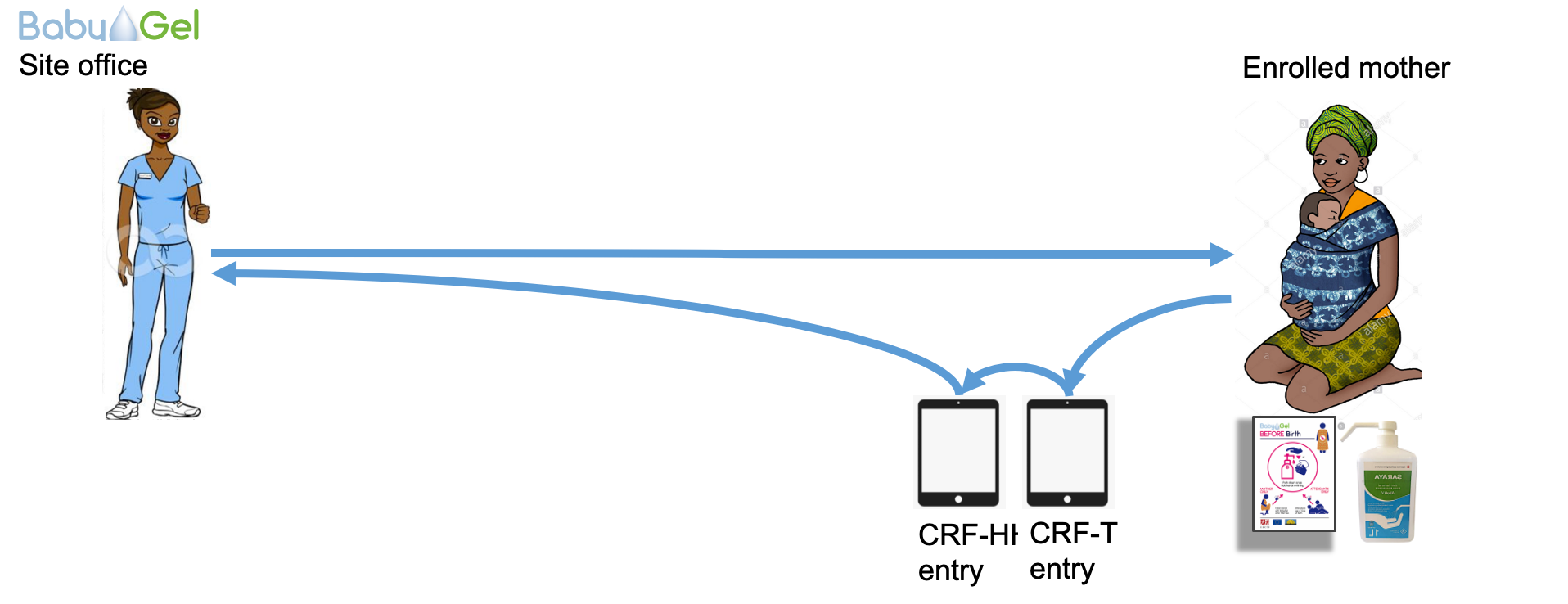
**

**Before you leave the office to VISIT 4:**

**Remember to Review:**

- Your notes from the last visit and prepare for your action points

**Before Training**

- Greet participant(s)

**Interview Introduction**

- Invite mother to select interview location, making sure the mother is comfortable and safe.
- Introduce the content of today: Hand Hygiene Questionnaire

**Interview:**

- Complete the HH-Questionnaire interviewing the mother

**BabyGel Training:**

- Note remaining amounts of ABHR (if any)
- If containers are missing, question missing containers
- Note poster location
- If poster is missing or in wrong location, inquire reasons

**Closing**

- Assure mother that she can keep all remaining materials
- Farewell

**After Training, near the house**

- Fill CRF T,
- Note household specific thoughts in your pad

**PHONE VISIT**

**
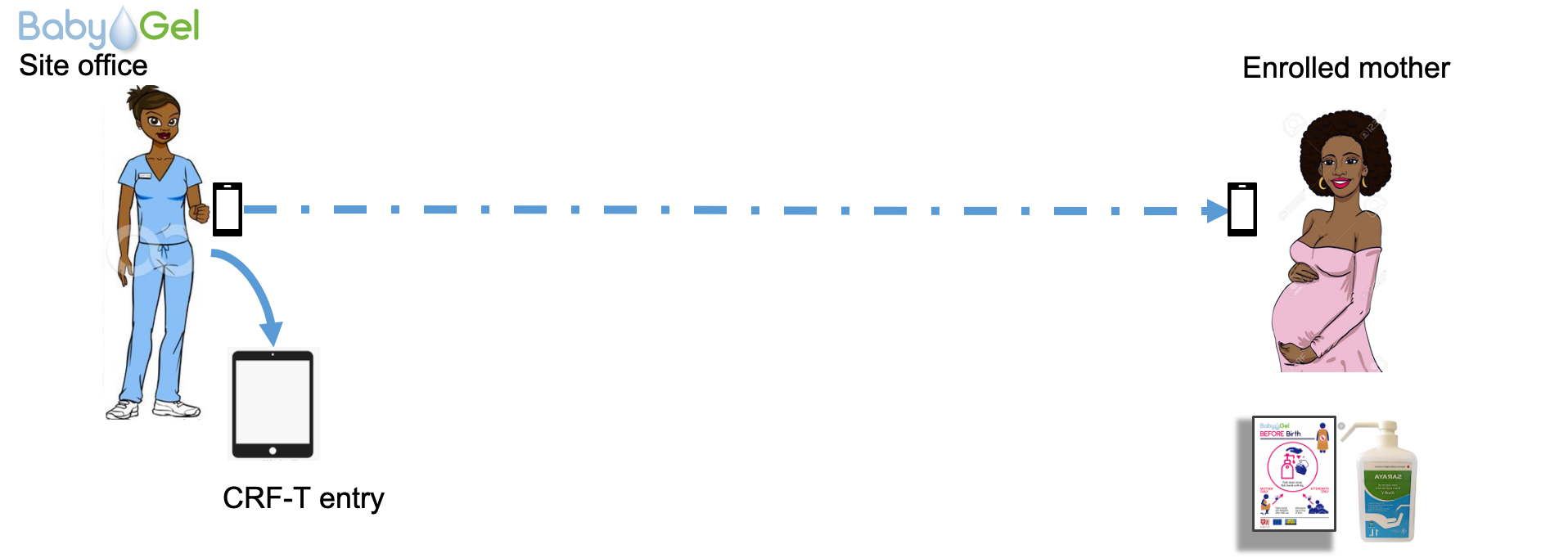
**

**Before you call the participant Remember to Review:**

- Your notes from the last visit and prepare for your action points
- Re-familiarize with CRF-T for phone interview

1. **Introduce the content of call, and give overview of Training Program**
   - 1. Today (10-15 min)
2. **Give mother opportunity to inquire about phone-based interview modality**
3. **State importance of ABHR at both times: before and after birth**
4. **Conduct interview, loosely following structure of CRF-T Phone**
5. **Any remaining questions?**
6. **Confirmation of emergency contacts and process at birth**
7. **Farewell**
8. **Fill CRF T on site, log GPS and times,**
9. **Note household specific thoughts for follow-up in your pad**

ABHR Safety:

- Does it burn easily?
- Can it be drunk?
- What if I spill it?
- Can I clean the baby with it?
- Can I clean the house with it?
- What if it burns?
- Can I cook with it?
- When does it go bad?
- Is it safe to eat with hands after ABHR use?
- What if someone drank it by accident?
- What do I do if ABHR gets into my baby’s or a child’s eyes?

ANHR Logistics:

- Can I get a refill?
- Where do I store it best?
- What do I do if I run out of ABHR?

ABHR use:

- Can my family, too, use it after toilet use?
- Why do I have to wait until the hands are dry after ABHR use?
- What else can I do to improve my hygiene (nails etc…)
- Do I get dry hands?
- Can I use it when I have a cut on my hands
- Can I use it do disinfect other cuts or wounds?
- Can I use it on the baby’s cord stump?
- Can I dilute ABHR to have more?

Cultural Context:

- Is it halal?

Health Questions:

- Can it harm the child?
- Can it harm my hands?
- Can I use it for other hygiene?

***Guide if mother not home***

Funnel included

Questionnaire (training) in the end?)
