# Supplementary material for: A cluster randomised trial to evaluate the effectiveness of household alcohol-based hand rub for the prevention of sepsis, diarrhoea, and pneumonia in Ugandan infants (the BabyGel trial): a study protocol
Source: Trials. 2023 Apr 17;24:279. doi: 10.1186/s13063-023-07312-1 (PMC10106319; doi:10.1186/s13063-023-07312-1)
Supplement: Supplementary file 4 — Additional file 4. [file 13063_2023_7312_MOESM4_ESM.pdf]

UNIVERSITY OF LIVERPOOL

EDCTP

SAfRI Sanyu Africa Research Institute

Trial ID					

ANC No					

Date of Form	D	D	M	M	Y	Y	Y	Y

Participant Initial			

BabyGel: A cluster randomised trial to evaluate the effectiveness of household alcohol-based handrub for the prevention of sepsis, diarrhoea and pneumonia in Ugandan infants

BabyGel

INSTITUTIONAL REVIEW BOARD
APPROVAL
15 JAN 2024
Valid Till
CURE
P.O. Box 903, Mbale (U) Tel: +256 4544 35356

PARTICIPANT CONSENT FORM

Public Title

BabyGel: A study to assess the usefulness of household sanitizer to prevent infections, diarrhoea and pneumonia in Ugandan babies.

Protocol version: ______ Date: ________________

If you are happy with each of the writings below, please put first letters of your names in each of the boxes or put a thumbprint and then sign and date at the bottom of the page.

1) I have read (or have been read) and understood the participant Information Sheet (Version:_____ Date:__________,) and asked any questions that I wanted to know. ☐

2) I am aware that it is my own choice whether to take part in this study and that I can stop taking part at any time without giving a reason. ☐

3) I agree to the research team to get information about my wellbeing from the hospital or from relatives or friends if the team has not been able to contact me directly for more than 2 weeks after agreed study visit date. ☐

4) I agree that photographs of my baby's umbilical cord stump may be taken and stored along with the information collected. I understand that my baby will not be found out, and the photo will include only the cord stump. ☐

5) If my baby becomes sick, I understand that blood, urine, or stool or cerebral spinal fluid (water from the baby's back) may be taken according to the best local accepted care by Ministry of Health to test. I will be told the results of all tests and given any ☐

treatment needed. I give permission for the results of tests and treatments to be included in the study.

6) I agree to allow information or results from this study to be used in future healthcare and/or medical research without me and my baby being noticed and without me needing to give any more permission. ☐

7) I understand that supervising organisations may look at the information collected in this study to ensure that the study is being done to appropriate standards ☐

8) After the study ends, I accept my information to be used by the research team for other studies. It may also be shared with the public and other researchers. It will not contain my name or any information that will allow them to identify me. ☐

9) I understand that information collected in this study will be taken out of Uganda for analysis. ☐

10) I agree to take part in the BabyGel study. ☐

Name of Participant	Date	Signature/thumbprint
______________________	____________	______________
Name of Witness (if applicable)	Date	Signature
______________________	____________	______________
Name of person taking consent	Date	Signature
______________________	____________	______________

TOP copy to be stored in the ISF, OTHER copy for the participant

Other organisations taking part in delivering this study

INSTITUTIONAL REVIEW BOARD
APPROVAL
15 JAN 2024
Valid Till
cure
P.O. Box 903, Mbale (U) Tel: +256 4544 35356
