# Supplementary material for: A cluster randomised trial to evaluate the effectiveness of household alcohol-based hand rub for the prevention of sepsis, diarrhoea, and pneumonia in Ugandan infants (the BabyGel trial): a study protocol
Source: Trials. 2023 Apr 17;24:279. doi: 10.1186/s13063-023-07312-1 (PMC10106319; doi:10.1186/s13063-023-07312-1)
Supplement: Supplementary file 5 — Additional file 5. [file 13063_2023_7312_MOESM5_ESM.pdf]

**BabyGel:** A cluster randomised trial to evaluate the effectiveness of household alcohol-based handrub for the prevention of sepsis, diarrhoea and pneumonia in Ugandan infants

BabyGel

## PARTICIPANT INFORMATION SHEET

**Public Title:** BabyGel: A study to assess the usefulness of household sanitizer to prevent infections, diarrhoea and pneumonia in Ugandan babies.

**Protocol Version No:_______ and Date: ____________**

**What you should know about this study:**

- You are being asked to join the BabyGel study.
- This sheet explains why the BabyGel study is being done and what will be needed from you and your baby
- Please read it carefully or have someone read it to you. Take your time to choose if you want to join. You can discuss it with your family or friends if you wish.
- In case you need more information, or have any questions, please ask the BabyGel research team or the regulatory authorities. Their contact details are provided at the end of this document.
- You are free to take part in this study. After we have given you the information about this study, you can choose to take part in this study or not. If you choose to take part in the study and at a later time you feel you should not continue, you may leave at any time. If you leave, your healthcare will not be affected at the existing health facilities. There will be no penalty if you choose to leave the study.



INSTITUTIONAL REVIEW BOARD
APPROVAL
Valid Till
15 JAN 2024
cure
P.O. Box 903, Mbale (U) Tel: +256 4544 35356

- You will be **given a copy of this information sheet** to continue referring to during the course of the study and it will be yours to keep.

**What is this study all about?**

When your baby is young, it can easily get an infection. The germs that cause the infection may come from the birth passage during delivery or be picked up from other people in the hospital or at home. When an infection is found, it can usually be treated with medicines. The response to medicines may vary depending on the type of medicine or the baby's body ability to fight infection. In healthcare, it is well known that it is better to prevent infection, and when it happens, try to treat it at the earliest opportunity.

Germs are usually carried on the hands of those who look after the baby. Dirty hands can carry germs to your baby from other people, from dirt or from animals. It is important to keep your hands clean when looking after your baby. The best way to keep your hands clean, is to wash them carefully with soap and enough clean water.

**What will the study involve?**

In this study we will assess whether using sanitizer for cleaning hands before the caretaker touches the baby and after using the toilet at household level will be useful in preventing infections in the baby. The sanitizer, we are using is called BabyGel and it will be used to clean hands by spraying the gel into the hands, rubbing the hands together until they dry. This will be done before touching the baby or after visiting the toilet. In the past, we tested this idea, but was in a small study in 10 villages around Mbale. The information we obtained from this small study has helped us to plan this larger study where we will look at babies born in some villages of Mbale and Budaka districts.

**Why is it important to do this study now?**

We planned this study before the onset of COVID-19 Pandemic. When Covid-19 was confirmed in Uganda, strict hand hygiene measures were put up including widespread use of sanitizer on the hands or washing using soap and clean water. However, the practice of handwashing is not yet routine, especially among men. The people sanitize to prevent COVID-19 due to orders of the president.

The COVID-19 response doesn't focus on preventing newborn infections. The Ministry of Health messages do not focus on handwashing nor sanitizing before touching the

INSTITUTIONAL REVIEW BOARD
APPROVAL
Valid Till
15 JAN 2024
cure
P.O. Box 903, Mbale (U) Tel: +256 4544 35356

baby, which leaves the baby at high chance of getting infections. Further, the study is taking place in villages where majority population have limited or no access to the sanitizer nor daily soap for effective hand hygiene to prevent newborn infections.

**Who will take part in this study?**

In this study, we will select 5932 women and their new born babies in 72 villages within Mbale and Budaka districts of Eastern Uganda.

**How can I take part in the study?**

First, you will need to answer some questions to see if you can take part in the study. This can be done by a research midwife at your home. If we think you can take part in this study, we will provide information to help you understand what the study is all about. This will help you choose whether you want to take part or not. If you choose to take part, you are still free to leave at any time and without giving a reason. If you choose to take part, you will be asked to sign an acceptance form to show that you freely offered to be part of this study.

**What will I need to do if I take part in the study?**

This study is made in such a way that mothers in some villages will receive the sanitizer while mothers in other villages will not. The villages have already been selected by chance and the research team will tell you about your village, should you choose to take part. The allocation as to whether mothers in any one village receive the sanitiser or not is done purely by chance. It is this arrangement that enables us, the researchers, to collect information without changing it. We have also given information about this study to your LC1 chairperson and got permission to carry out this study in this village. So, if you agree to join, you will receive exactly the same treatment as other pregnant women in your village. Women from one village may not be the same as those from another village based on whether or not they are given the sanitizer. This is done because we do not know at this point whether giving and using BabyGel will help prevent infection in babies or not.

We will ask you some questions about any past pregnancies and your current pregnancy. We will then give you some teaching on how and when to hand wash to prevent infections in you and your baby.

If you agree to be in the study, the research team will then tell you which pack you will receive. They will record your details, assess your resources including water and depression status during the pregnancy.



INSTITUTIONAL REVIEW BOARD
APPROVAL
Valid Till 15 JAN 2024
cure
P.O. Box 903, Mbale (U) Tel: +256 4544 35356

In all villages, we will give all mothers the things needed for delivery as stated in the Uganda Ministry of Health guidelines, whether you will get Sanitizer or not. We will give a Maama kit having things for use at the time of delivery, a small bar of soap and a tube with a gel called Umbigel (chlorhexidine) for putting on your baby's cord.

In a half of the villages, mothers will also receive BabyGel as well as the Maama kit from the study team member. If you are given BabyGel for handwashing, you will use it every time before touching the baby, both at home and away from home. We will give you guidance on how and when to use it. It will mean cleaning your hands every time before touching the baby with the BabyGel until 3 months after childbirth. You will also be told the contents of the BabyGel.

The BabyGel will come in a box having a full 5 litre jerrycan, a full 1 litre bottle for use at home and a small empty bottle to be used by mother when moving away from home. A study team member (trainer) will deliver the 1-litre bottle at time of recruitment for use before birth and the rest of the study pack delivered on the 3rd day after birth.

**How will I be followed up in the study?**

We would like to check on your health and that of your baby from around 34 weeks of your pregnancy until 12 weeks after you have given birth. A research midwife will take contact details from you at the time of joining the study. This information will be for this study purpose only and will not be shared with any other person who is not part of the study. The study team will keep in touch with you. This may be by telephone, through your village health Team (VHT) member, or at a home visit. Keeping in contact is important so that we know when you give birth and can check on you and your baby's health.

In Uganda, the Ministry of Health advises all mothers to deliver in a health facility or hospital. Once you give birth and have gone back to your home, you should tell your VHT or research midwife as soon as possible. He/she will then tell a research midwife, who is a member of our study team, who will visit you to get some information about your delivery and your baby. You can also directly inform a member of our study team assigned to you. The research midwife will try to visit you within 2 days after birth.

A research midwife will always keep in touch with you to assess your health and that of your baby. During the visit, they will ask you some questions and may take a photo of your baby's umbilical 'cord stump'. They will remind you to go to the nearby health centre if your baby gets sick. The research midwife will sometimes assess you by

INSTITUTIONAL REVIEW BOARD
APPROVAL
Valid Till 15 JAN 2024
cure
P.O. Box 903, Mbale (U) Tel: +256 4544 35356

telephone and sometimes in your home. If you have no telephone contact available (through a family or friend), then all check-ups will be in your home. More visits may be done if you or your baby becomes sick.

The study will end when your baby is 3 months old. At this point, the research team will do of the last visit to assess you and your baby in your home. This information will help to tell us why babies become sick and how infection is spread.

**What should I do if my baby gets sick?**

It is important for you to inform us **if your baby becomes sick.** You can contact us through the study telephone number. You will not be charged airtime for calling this number when your baby is sick. You can also beep/flash a mobile telephone of the research team or the VHT.

**If your baby becomes sick**, you will be referred to the nearby health centre or the Mbale Regional Referral Hospital Neonatal Unit (MRRH NNU), a ward for sick newborn babies, for treatment of their condition. The treatment your child will receive is not part of this research but will be the acceped treatment by the Uganda Ministry of Health. If you go to the health centres, doctors will check your baby and either treat your baby there or refer your baby to MRRH-NNU if necessary. Transport to MRRH-NNU will be paid for by the research study. You or your baby will be given free treatment and support when your baby stays in hospital.

**If your baby is admitted to MRRH-NNU** with an infection. The baby will be assessed and treated by the neonatal team in the usual way. We will ask you questions about your pregnancy and the baby. A doctor will assess the baby fully and record all signs of infection that the baby might have.

Sometimes when babies are admitted and treated for infection, some blood and cerebrospinal fluid (water around the baby's brain and spinal cord that is drawn by pushing a needle in the lower back) are taken from the baby and sent to the laboratory for tests to find the exact germ causing the illness. We will offer to do these tests for you, to help find the cause of the infection in your baby. We will include tests for malaria and HIV. Remember, HIV testing has been recommended for all patients who go to the government health facilities seeking for treatment. We will ask for your permission before we do this test.

If you agree, the doctor will draw blood from the baby and take a cerebrospinal fluid (water from the baby's back). These samples will be sent to the laboratory in Mbale



INSTITUTIONAL REVIEW BOARD
APPROVAL
Valid Till 15 JAN 2024
cure
P.O. Box 903, Mbale (U) Tel: +256 4544 35356

for tests to try and find out the exact germs that might be causing the baby's illness. We will give these results to your baby's doctor to help improve their care.

If your baby has no sign of sickness, then s/he will remain with you at home unless there are other concerns. You should see a health worker again if your baby gets worse or develops any new problems.

**Will I be invited to participate in any other studies?**

If you join this study, you may also be invited to join other small studies to collect extra information of use for research that is not part of this main study. In such cases, you will be given further information. Your decision to join other studies would not affect your care in this main study.

**Do I have to take part?**

No. It is up to you to choose whether to join the study. You are free to take part in this study and leave at any time. This will not affect the care you and your baby receive.

**Can my participation in this study be stopped without my consent?**

You may be taken off the whole study without your acceptance if the study is cancelled by the study funder. the Sponsor of the study, regulatory authorities in Uganda, or the local research ethics committee (REC).

**What will happen if I want to leave this study?**

If you choose to take part, you are still free to leave the study at any time and without giving a reason. However, sharing your reason for leaving the study would be helpful. This information may help us to do research studies in the future. If you leave, we will still keep information relating to the treatment given to you, as this is important for the study. Before you leave, you will be asked whether we can still continue using the information we have already collected about you and your baby for research purposes or not. Should you decide to leave the study you and your baby will receive usual care given at the health centres.

**What will happen if I choose not to take part in this study?**

You are free to take part or not take part in this study. If you do not wish to join the study, the research team will advise you to continue your regular antenatal care at the nearby health centre according to the normal accepted treatment.

**What are the possible risks of taking part in this study?**

INSTITUTIONAL REVIEW BOARD
APPROVAL
Valid Till
15 JAN 2024
cure
P.O. Box 903, Mbale (U) Tel: +256 4544 35356

If you are in a village receiving the BabyGel, most people can use BabyGel without any problems at all. However, if you have a cut or wound on your hand, then it may pain when you use the hand rub. Sometimes, people react to the sanitizer and their skin can become dry, red or itchy. If this happens, then you should stop using it as soon as possible and inform the research team.

The BabyGel also needs to be handled with care. It contains a form of alcohol, so it can cause a fire easily. It must be kept away from fires and prolonged sunshine. When still wet on hands, it can easily catch fire. Do not go near fire when the hands are still wet with the sanitizer. It can make you sick if you drink it and should be kept out of reach of children or animals.

**Are there any benefits for taking part in this study?**

By taking part in this study, you may benefit from extra attention to details of your care and clinical care for your baby.

If you are in a village receiving BabyGel, you may like to know that this BabyGel is already commonly used for hand cleaning by doctors and nurses in hospitals, by people in some homes and in the surrounding places as sanitizer. Using BabyGel for this study should keep your hands very clean and may reduce infections in both you and your baby. Many users also like the feel of the sanitiser on their hands.

**Will I receive any money for taking part in this study?**

You will not receive any money for taking part in this study. However, if your baby needs to go to hospital during the study, you will receive an allowance for the transport costs to move to the hospital and support when your baby stays in hospital. This will be in case of sickness until the baby is 3months of age. After which, the study will not take care of these costs.

**What if I or my baby gets a problem while taking part in the study?**

If you have a concern about any part of this study, you should speak first to the researchers who will do their best to answer your questions. If you are still unhappy and wish to complain, you can do this via Dr Benon Wanume, Director of the Sanyu Africa Research Institute (telephone 0772479249 and email wanumeb@hotmail.com).

If something goes wrong and you get injured during the research, and this is due to the study team's mistake, then you may be able to take action against the University of Liverpool in the UK.



INSTITUTIONAL REVIEW BOARD
APPROVAL
Valid Till
15 JAN 2024
cure
P.O. Box 903, Mbale (U) Tel: +256 4544 35356

## What happens to me after the study ends?

Joining the BabyGel study does not affect your treatment or that of your baby at the end of the study. At the end of the study, you and your baby will continue with the national health care programmes including immunisation.

## Will my taking part in the study be kept confidential?

Yes. We will follow best ethical and legal practice and all information about you will be handled secretly. All personal information with your name attached to it will be kept strictly a secret and will only be looked at by members of the research team (or by authorities checking that the trial is being carried out correctly). Any information about you, that is shared beyond that, will have your name removed so that others will not be able to link it with you personally.

## What will happen with the results of the study?

By taking part in this study, you will be helping us to study ways to stop infections in the first three months of the baby's life. If the study shows that BabyGel is better than accepted care, this will be an important finding. It will help to improve the prevention of newborn infections, and this may be of use to you and many other people like you in the future.

Three local patient groups at each Health Centre will meet to comment on the study process and early results from the study. They will be invited to assess the results from the BabyGel study together with members of the patient and public involvement steering group. They will share their stories with African Sepsis Alliance, "Every patient in Africa has a right to survive campaign" handwashing campaign. The results will also be shared locally in a stakeholders' meeting, local radio stations, national and international conferences, and shared widely in recognised health journals, websites and on social media.

## Who is organising and funding the study?

The study is being run by the University of Liverpool in the UK and the Sanyu Africa Research Institute in Mbale. Other partners on the study include Liverpool School of Tropical Medicine (LSTM) and the University of Exeter (UoE) in the UK, the University of Bergen (UiB) in Norway, Makerere University, Busitema University, Mbale Regional Referral Hospital (MRRH), and Ministry of Health in Uganda. The study is funded by the European Developing Countries Clinical Trials Partnership (EDCTP) in Europe.

INSTITUTIONAL REVIEW BOARD
APPROVAL
Valid Till
15 JAN 2024
cure
P.O. Box 903, Mbale (U) Tel: +256 4544 35356

**Who has reviewed the study?**

This study has been checked and approved by the University of Liverpool, the regional Norwegian Government Ethics Committee and Cure Children's Hospital of Uganda Research Ethics Committees. The Uganda National Council for Science and Technology has also approved this study. These independent committees make sure that the study is high quality, does not put you at any risk and that any information you give to the research team will be treated secretly. If you are not satisfied with the study, you can contact Dr. Oketchi Humphrey, the Chairperson CURE Children's Hospital of Uganda Research Ethics Committee (CCHU-REC) on Telephone number 0779372445.

**Whom can I contact in the study?**

If you would like to speak to a member of the research team, please contact Martin Chebet on +256774038006

**Thank you for taking the time to read or listen to me.**

Other organisations taking part in delivering this study

INSTITUTIONAL REVIEW BOARD
APPROVAL
Valid Till 15 JAN 2024
cure
P.O. Box 903, Mbale (U) Tel: +256 4544 35356
