# Supplementary material for: A cluster randomised trial to evaluate the effectiveness of household alcohol-based hand rub for the prevention of sepsis, diarrhoea, and pneumonia in Ugandan infants (the BabyGel trial): a study protocol
Source: Trials. 2023 Apr 17;24:279. doi: 10.1186/s13063-023-07312-1 (PMC10106319; doi:10.1186/s13063-023-07312-1)
Supplement: Supplementary file 6 — Additional file 6: Picture 1. Components of a Maama birth kit given to pregnant women in the BabyGel Trial. Picture 2. Alcohol Based Handrub Package given to pregnant women in the intervention arm of the BabyGel Trial. [file 13063_2023_7312_MOESM6_ESM.pdf]

BabyGel Protocol Pictures

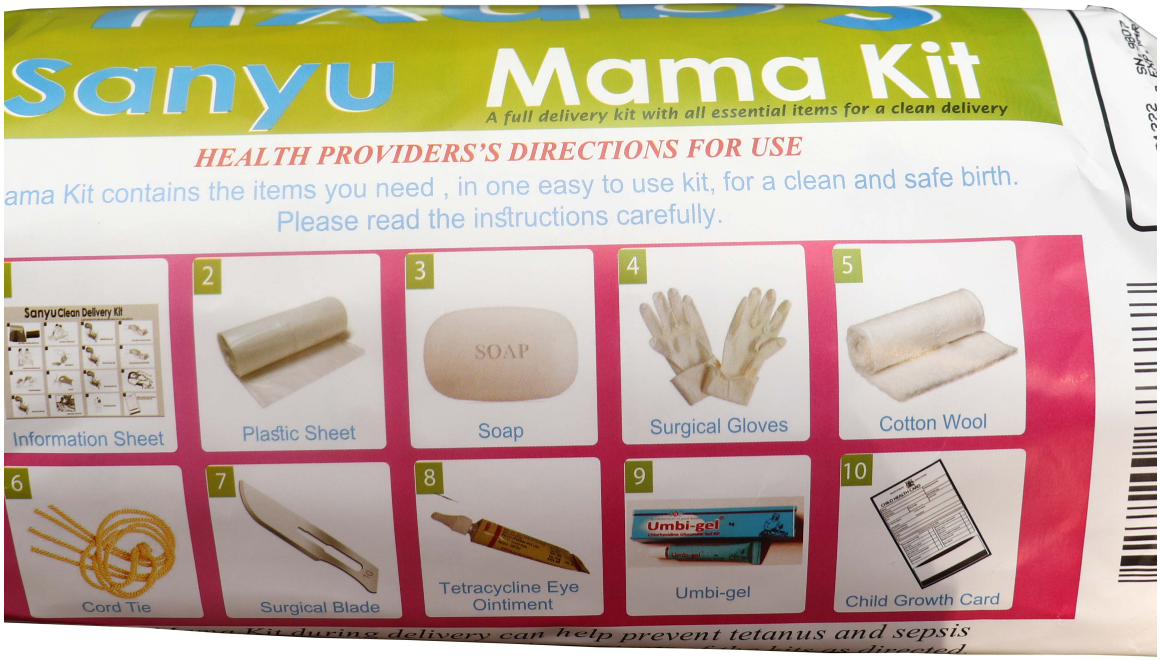

Picture 1: Components of a Maama birth kit given to pregnant women in the BabyGel Trial

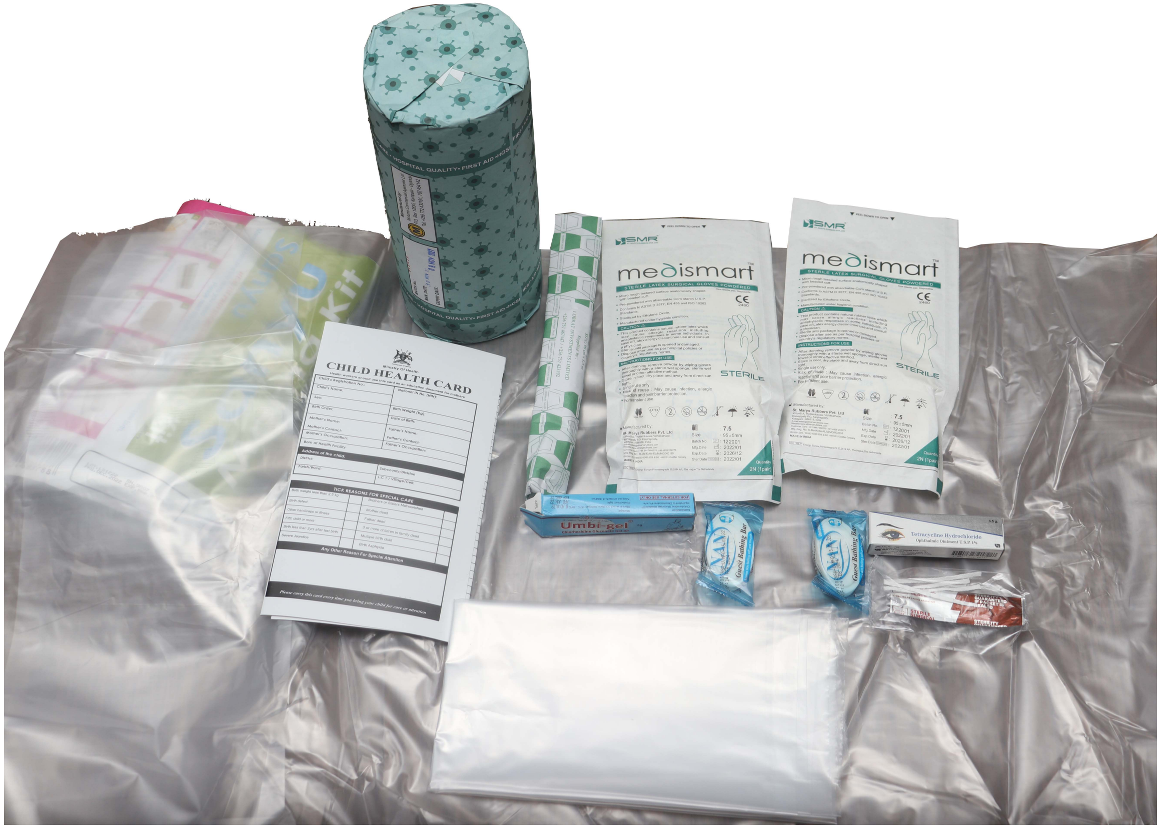

Picture 1: Components of a Maama birth kit given to pregnant women in the BabyGel Trial

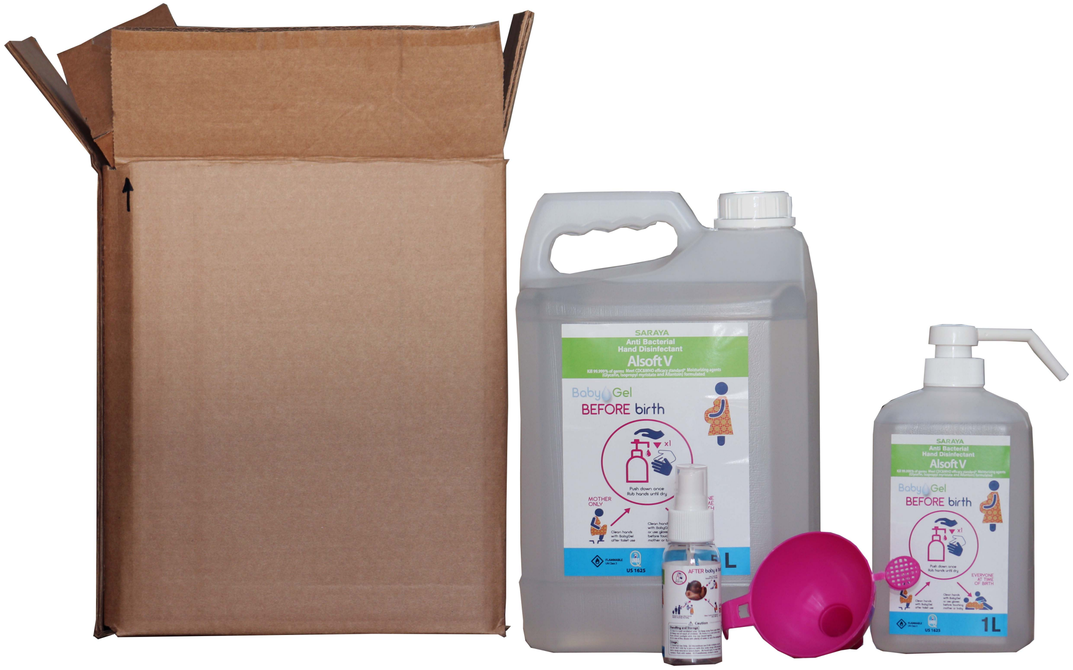

Picture 2: Alcohol Based Handrub Package given to pregnant women in the intervention arm of the BabyGel Trial
